# HLA-E/peptide complexes differentially interact with NKG2A/CD94 and T cell receptors

**DOI:** 10.1093/jimmun/vkae068

**Published:** 2025-03-14

**Authors:** Linda Voogd, Remco L van den Broek, Marjolein van Wolfswinkel, Kees L M C Franken, Paula Ruibal, Willem Jespers, Judith Leitner, Peter Steinberger, Gerard J P van Westen, Tom H M Ottenhoff, Simone A Joosten

**Affiliations:** Leiden University Center for Infectious Diseases, Leiden University Medical Center, Leiden, The Netherlands; Division of Medicinal Chemistry, Leiden Academic Centre for Drug Research, Leiden University, Leiden, The Netherlands; Leiden University Center for Infectious Diseases, Leiden University Medical Center, Leiden, The Netherlands; Leiden University Center for Infectious Diseases, Leiden University Medical Center, Leiden, The Netherlands; Leiden University Center for Infectious Diseases, Leiden University Medical Center, Leiden, The Netherlands; Division of Medicinal Chemistry, Leiden Academic Centre for Drug Research, Leiden University, Leiden, The Netherlands; Centre for Pathophysiology, Infectiology and Immunology, Institute of Immunology, Medical University of Vienna, Vienna, Austria; Centre for Pathophysiology, Infectiology and Immunology, Institute of Immunology, Medical University of Vienna, Vienna, Austria; Division of Medicinal Chemistry, Leiden Academic Centre for Drug Research, Leiden University, Leiden, The Netherlands; Leiden University Center for Infectious Diseases, Leiden University Medical Center, Leiden, The Netherlands; Leiden University Center for Infectious Diseases, Leiden University Medical Center, Leiden, The Netherlands

**Keywords:** HLA-E, NKG2A/CD94, peptide/receptor interactions, T cell receptor

## Abstract

The virtually monomorphic antigen presentation molecule HLA-E can present self- and non-self peptides to the NKG2A/CD94 co-receptor inhibitory complex expressed on natural killer (NK) cells and to T cell receptors (TCRs) expressed on T cells. HLA-E presents self-peptides to NKG2A/CD94 to regulate tissue homeostasis, whereas HLA-E restricted T cells mediate regulatory and cytotoxic responses toward pathogen-infected cells. In this study, we directly compared HLA-E/peptide recognition and signaling between NKG2A/CD94 and 2 HLA-E restricted TCRs that can recognize self-peptides or identical peptide mimics from the viral UL40 protein of *cytomegalovirus* using position substituted peptide variants. We show that position 7 is critical for interaction with NKG2A/CD94, whereas position 8 is important for interaction with the TCRs. The Arginine at position 5 of these peptides is an essential residue for recognition by both receptors. Thus, NKG2A/CD94 and TCRs have different requirements for recognition of peptides presented in HLA-E.

## Introduction

HLA-E is a non-classical antigen presentation molecule with 2 functional alleles expressed in humans, called HLA-E*01:01 and HLA-E*01:03.^1^ The single amino acid difference between these alleles is located outside the peptide binding groove suggesting that both alleles have a comparable peptide binding repertoire, although differences in cell surface stability and peptide affinity have been reported.^2^ Both HLA-E alleles are expressed in balanced equilibrium among the world population.^3^ HLA-E can present nonameric peptides derived from leader sequences of classical HLA-I molecules, called self-peptides or in short VL9-peptides, pathogen-derived peptides and tumor-derived peptides.^4–12^ HLA-E is an attractive target for vaccination to induce population wide protection because of its virtually monomorphic character.^13^^,^^14^

Initial studies identified peptide positions 2, 7, and 9 as the primary anchor residue positions for HLA-E, which were restricted to the hydrophobic residues Methionine (position 2) and Leucine (position 7 and 9).^15^ Recently, we used a more extensive peptide library to optimize the peptide binding algorithm for HLA-E.^7^ We showed that HLA-E in addition to Leucine also prefers larger hydrophobic residues, such as Tryptophan, at primary anchor residue position 9. In addition, position 7 can also accommodate Proline and Alanine residues reflecting more flexibility than previously anticipated.^7^ Smaller residues, such as Alanine and Threonine, are preferred adjacent to these anchor positions, possibly facilitating binding of larger residues at the anchor positions. The peptide binding motifs for both HLA-E alleles revealed that binding to HLA-E*01:01 is more permissive than to HLA-E*01:03, especially at positions 5 and 7.^7^

The first described biological role for HLA-E is the interaction with the NKG2A or NKG2C/CD94 co-receptor complex expressed on natural killer (NK) cells.^16^^,^^17^ These co-receptors recognize HLA-E/VL9-peptide complexes and can inhibit (NKG2A/CD94) or activate (NKG2C/CD94) NK cell-mediated lysis of the presenting cell. NKG2A/CD94 has a higher affinity for HLA-E/VL9-peptide complexes than NKG2C/CD94 but both receptors have a comparable HLA-E/VL9-peptide recognition motif.^18^ The VL9-peptides are generated in the Endoplasmic Reticulum (ER) from nascent classical HLA-I molecules via the proteolytic activity of various signal peptide peptidases and are loaded onto HLA-E in the ER membrane.^13^  *Cytomegalovirus* (CMV) can present identical VL9-peptide mimics from the UL40 protein onto HLA-E to prevent lysis of the infected cell via interaction with NKG2A/CD94.^19^ In contrast, defects in the antigen presentation machinery results in improper HLA-E peptide loading, which activates NK cell-mediated lysis due to abrogated interaction with NKG2A/CD94. The sequence of these VL9-peptides depends on the classical HLA-I allele and are variations of the sequence VMAPRTLLL.^20^ Differences between VL9-peptides are located at positions 2, 7, and 8. CD94 directly interacts with the peptide at positions 5, 6, and 8, and NKG2A interacts with the α2 helix of HLA-E, not directly with the peptide.^21^ Position 7 is also involved in NKG2A/CD94 recognition but indirectly as it positions other residues for engagement with the receptor. The peptide binding affinity for HLA-E and the strength of the NKG2A or -C/CD94 signal upon recognition of HLA-E/VL9-peptide complexes depends strongly on the VL9-peptide sequence.^20^

Next to NKG2A or -C/CD94, peptides presented in HLA-E can also be recognized by T cell receptors (TCRs). HLA-E restricted peptides that can be recognized by TCRs are derived from several pathogens, such as CMV, *Mycobacterium tuberculosis* (*Mtb*), *Salmonella Typhimurium*, HIV and *Epstein-Bar* virus, or from self-peptides.^4–11^ HLA-E is virtually monomorphic, therefore HLA-E restricted T cells belong to the family of donor unrestricted T cells (DURTs).^22^ TCR-αβ sequences that can recognize HLA-E/peptide complexes were expected to be invariant, but we recently demonstrated that HLA-E/*Mtb* restricted TCRs are highly diverse within and between individuals.^23^ The canonical binding mode is similar as for conventional TCRs, that is, the TCR-β chain interacts with the α1 helix and the TCR-α chain with the α2 helix of HLA-E.^24^ The interaction on a molecular level involves an intricate network of non-covalent interactions, heavily dependent on the peptide sequence and the TCR-α and -β chains. A previous study on the well-characterized TCR clone KK50.4 in complex with the VMAPRTLIL peptide from CMV showed that peptide position 8 was the main contact residue with the TCR.^25^

Altogether, NKG2A/CD94 and TCRs, two structurally and functionally different receptors, can recognize HLA-E/peptide complexes. Here, we aim to decipher the differences in preferred peptide sequences on a residue level for recognition and signaling by the NKG2A/CD94 complex and TCRs. To this end, variants were synthesized of the HLA-E presented peptide sequences VMAPRTLIL, VLAPRTLLL and several *Mtb* peptides and were tested for direct comparison of receptor recognition and signaling. TCRs included were the TCR KK50.4 clone recognizing the VMAPRTLIL peptide and a TCR recognizing VL9-peptides that was identified in our previous study, named TCR 6.^23^^,^^25^ We show that the main difference between NKG2A/CD94 and TCRs for interacting with HLA-E/peptide complexes is located at position 7 and 8 of the peptide and show that position 5 is crucial for recognition by both receptors. Thus, NKG2A/CD94 and TCRs differentially recognize HLA-E/peptide complexes, with other preferred residues at peptide positions 7 and 8.

## Material and methods

### Peptide sequences

Amino acid sequences of the peptide variants are shown in Table S1 and are summarized in Fig. S1. Peptides are variations of the published wildtype sequences UL40 (VMAPRTLIL), VL9 (VLAPRTLLL), Mtb44 (RLPAKAPLL), Mtb34 (VMTTVLATL) and Mtb55 (VMATRNNVL) and were synthesized by Peptide 2.0 Inc. with 95% purity.^4^^,^^7^

### UV-mediated peptide exchange reaction and detection by sandwich ELISA assay

Peptide variants were tested for HLA-E*01:03 binding affinity using the previously developed UV-mediated peptide exchange coupled to ELISA assay for HLA-E.^26^ Briefly, HLA-E*01:03 monomers folded with a UV-sensitive peptide were exchanged with the peptide variants by exposure to UV light for 1 h at 4°C. Exchanged HLA-E monomers were added to anti-human HLA-E monoclonal antibody (clone 3D12, BioLegend) coated 96-well half-area ELISA microplates (Greiner-Bio). HLA-E/peptide complex formation was evaluated by addition of horseradish peroxidase (HRP)-conjugated anti-β2m antibodies (Thermo Fisher) and the signal was amplified using HRP-coupled goat anti-rabbit IgG antibodies (Dako). Read-out was performed via measuring the absorbance at 450 nm using the SpectraMax i3x Reader (Bio-Rad). Peptide affinity was calculated by subtracting the background absorbance (no peptide condition) from the test condition and was normalized to the absorbance of the positive control condition (VMAPRTLIL or VLAPRTLLL) minus background absorbance and is represented as a signal to positive (S/P) ratio. Cutoffs to classify as non-binders, binders or strong binders were defined previously.^7^

### Transfections and transductions

Human 293-based Phoenix GALV packaging cells were transfected as described previously with 2.6 µg MP71flex retroviral vector containing the variable TCR-αβ and CDR3-αβ sequences from a TCR recognizing VL9-peptide sequences (obtained from our previous RNA-seq analysis on HLA-E*01:03 tetramer sorted CD8^+^ T cells) or from the published KK50.4 T cell clone recognizing the UL40-derived peptide sequence VMAPRTLIL from CMV (Table 1).^23^^,^^25^ The MP71flex vector contains several unique cut-sites which allows the exchange of variable TCR-αβ domains into the vector and contains codon-optimized murine TCR-αβ constant domains to increase the expression and pairing of the introduced TCR-αβ chains.^27^^,^^28^ Transduction of viral supernatant into JE6.1 CD8^+^ Jurkat triple parameter reporter (TPR) cells was performed as described previously.^23^ Expression of the TCR constructs was evaluated by staining with hamster anti-murine constant TCR-β APC-Cy7 (BioLegend) and mouse anti-human CD3 BV510 (BioLegend).

**Table 1.** Sequences of TCR 6 and TCR KK50.4 that were transduced into JE6.1 CD8^+^ TPR cells.

	TRAV	TRAJ	CDR3α	TRBV	TRBJ	CDR3β
**TCR 6**	TRAV2	TRAJ20	CAVGLGDYKLSF	TRBV20-1	TRBJ1-1	CSARGVAEGWNTEAFF
**TCR KK50.4**	TRAV26-2	TRAJ37	CIVVRSSNTGKLIF	TRBV14	TRBJ2-3	CASSQDRDTQYF

### Co-culture of TCR transduced Jurkat JE6.1 CD8^+^ TPR cells with peptide-loaded K562-red fluorescent protein (RFP)-β2m-HLA-E*(Y84C) cells

In total, 20.000 K562-RFP-β2m-HLA-E*(Y84C) cells (from Dr P. Steinberger^29^) were loaded with 100 µM peptide for 20 h at 23°C in 50 µl Roswell Park Memorial Institute (RPMI) 1,640 medium (Gibco) + 10% Fetal Bovine Serum (FBS) (Capricorn) + 1% Penicillin/Streptomycin (P/S) (Gibco) in a 96-well round bottom plate (Greiner-Bio). Also, 50.000 TCR-transduced Jurkat cells were subsequently added to the peptide-loaded K562 cells in 100 µl final volume and were co-cultured for 18 to 24 h at 37°C, 5% CO_2_. Stimulation with K562 cells loaded with peptides VMAPRTLIL or VLAPRTLLL were included as positive controls for the triple reporters. Stimulation with unloaded K562 cells was included as negative control for the triple reporters. Co-cultures were washed in Phosphate Buffered Saline (PBS) (Fresenius Kapi) supplemented with 0.1% bovine serum albumin (BSA) (Sigma-Aldrich) (PBS/0.1% BSA), stained for 30 min at 4°C in the dark with hamster anti-murine TCR-β APC-Cy7 (BioLegend) and mouse anti-human CD3 BV510 (BioLegend) (1:100 each) in PBS/0.1% BSA to identify the TCR expressing Jurkat population. Cells were washed in PBS/0.1% BSA, fixated in 1% paraformaldehyde (PFA) (LUMC pharmacy), washed again in PBS/0.1% BSA and acquired on a BD LSRFortessa™ (BD Bioscience). Samples were analyzed with FlowJo v10.8.1 using the gating strategy shown in Fig. S3A and further analysis was done in GraphPad Prism v10.2.3. Signal of reporters in stimulated samples was calculated by subtracting the signal in the negative control condition from the stimulated condition.

### Co-culture of JE6.1 Jurkat NKG2A/CD94 reporter cells with peptide-loaded K562-S(aCD3)-RFP-β2m-HLA-E*(Y84C) cells

The principle of this co-culture assay is described in Battin et al. 2022, and cells were obtained from Dr P. Steinberger.^29^ Briefly, interaction of membrane bound anti-CD3-scFv expressed on K562 HLA-E cells with the CD3/TCR complex expressed on JE6.1 NKG2A/CD94 cells activates the single reporter construct (NF-kB coupled to eGFP). Interaction of NKG2A/CD94 with the HLA-E/peptide complex reduces the reporter signal and this reduction represents the inhibitory signal (Fig. S3B for schematic overview). 20.000 K562-S(aCD3)-RFP-β2m-HLA-E*(Y84C) cells were loaded with 400 µM peptide for 20 h at 23°C in 50 µl RPMI + 10% FBS + 1% P/S in a 96-well round bottom plate (Greiner-Bio). Unbound peptides were washed away, and peptide loaded K562 HLA-E cells were cultured with 50.000 JE6.1 NKG2A/CD94 reporter cells in 100 µl final volume for 18 to 24 h at 37°C, 5% CO_2_. The reporter signal induced by unloaded K562 stimulator cells was used as positive control for the reporter signal, whereas stimulation with K562 cells loaded with VMAPRTLIL or VLAPRTLLL was used to achieve maximal inhibition of the reporter. Co-cultures were washed in PBS/0.1% BSA, fixated in 1% PFA, washed again and acquired on a BD LSRFortessa™ (BD Bioscience). Samples were analyzed using FlowJo v10.8.1 using the gating strategy shown in Fig. S3B and further analysis was performed in GraphPad Prism v10.2.3. Percentage inhibition of the reporter for each peptide was calculated using the following formula:


$$\%\;\mathrm{inhibition}\;\mathrm{reporter}\;=100*\;(\frac{\mathrm{gMFI}\;\mathrm{reporter}\;\mathrm{peptide}\;\mathrm{variant}\;-\mathrm{gMFI}\;\mathrm{reporter}\;no\;\mathrm{peptide}}{\mathrm{gMFI}\;\mathrm{reporter}\;\mathrm{wildtype}\;\mathrm{peptide}-\mathrm{gMFI}\;\mathrm{reporter}\;no\;\mathrm{peptide}})$$

### Staining of TCR and NKG2A/CD94 expressing cells with thermal exchanged HLA-E*01:03 tetramers

Thermal exchanged HLA-E*01:03 tetramers (TMs) were produced in-house as described previously.^30^ Briefly, HLA-E*01:03 monomers folded with a thermal sensitive peptide (VLRPGGHFAA) were exchanged with the peptide variants for 1 h at 30°C. Correct folding of the monomers was confirmed with HPLC and binding to LILRB1 expressing K562 cells, as described previously.^26^ Thermal exchanged biotinylated monomers were subsequently multimerized via coupling to streptavidin labelled phycoerythrin (PE) to obtain PE-labeled TMs. A minimum of 50.000 TCR-transduced Jurkat cells, K562 cells expressing NKG2A/CD94 or K562 cells expressing LILRB1 were stained with the thermal exchanged HLA-E TMs 1:4 in 30 µl PBS/0.1% BSA for 30 min at 37°C in the dark. Cells were washed in PBS/0.1% BSA, fixated in 1% PFA for 10 min at RT and washed again. TCR-transduced Jurkat cells were further stained with hamster anti-murine TCRβ APC-Cy7 (BioLegend) and mouse anti-human CD3 BV510 (BioLegend) (1:100 each) in 50 µl PBS/0.1% BSA for 30 min at 4°C in the dark. Cells were washed, fixated in 1% PFA and washed again. Samples were acquired on a BD LSRFortessa™ and analyzed using FlowJo v10.2.3.

### Principal component analysis (PCA)

Correlation analyses between assays were performed for both the combined VMAPRTLIL and VLAPRTLLL peptide set, as well as the VMAPRTLIL peptide set separately. The following assays were included in the correlation analyses for VLAPRTLLL: NKG2A/CD94 HLA-E*01:03 TM staining, NKG2A/CD94 reporter inhibition and TCR 6 HLA-E*01:03 TM staining. Additionally, for the VMAPRTLIL peptide set, the TCR KK50.4 HLA-E*01:03 TM staining and TCR KK50.4 reporter activation assays were included. The assay measurements were standardized and reduced to two principal components using the ‘StandardScaler’ and ‘PCA’ functions from scikit-learn (version 1.4.0).

## Results

### Binding affinity of variants to HLA-E*01:03 is residue dependent

**Figure 1.**
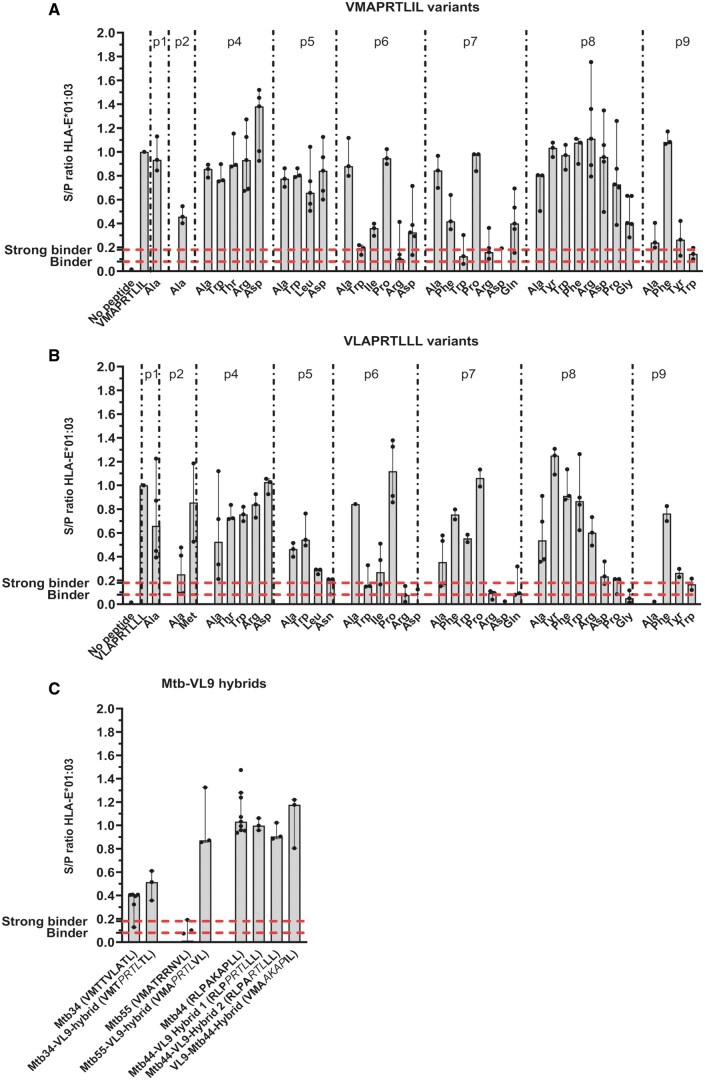
Binding of peptide variants and Mtb-VL9 hybrids to HLA-E*01:03. All peptides were evaluated for peptide binding affinity using the previously developed UV-mediated peptide exchange coupled to ELISA assay for HLA-E.^7^ (A) Affinity of the VMAPRTLIL variants for HLA-E*01:03. (B) Affinity of the VLAPRTLLL variants for HLA-E*01:03. (C) Affinity of the Mtb-VL9 hybrid peptides for HLA-E*01:03. The sequences of the hybrid peptides are shown on the X-axis and the residues that are changed relative to the wildtype sequence are shown in italics. The residue substitutions at each position are shown on the *X*-axis in (A) and (B). The *Y*-axis shows the affinity for HLA-E*01:03, displayed as a signal to positive ratio (S/P) in (A–C). The S/P ratio is a relative measure of the affinity of the peptide variant relative to the positive control peptide (VMAPRTLIL in A and VLAPRTLLL in B). Dotted lines indicate the threshold for defining peptides as binders (>0.08) or good binders (>0.18). These values were defined previously.^7^ Peptides were tested at least two times and bars represent the median S/P ratio with 95% confidence interval. Peptide sequences are provided in Table S1.

VMAPRTLIL and VLAPRTLLL variants, as well as HLA-E specific Mtb-peptides substituted with residues from VL9-peptides (Mtb-VL9 hybrids), were evaluated for peptide binding affinity to HLA-E*01:03 (Fig. 1A–C). These VMAPRTLIL and VLAPRTLLL variants contain residues introducing a different charge or size relative to the wildtype sequence (Table S1 for sequences). The binding affinity is calculated as a signal to positive (S/P) ratio.^7^ The S/P thresholds to define binders or strong binders are 0.08 and 0.18, respectively, as published previously, and are visualized with dotted lines in Fig. 1A–C.^7^ VLAPRTLLL variants with Arg and Asp at position 6 or 7 could not bind to HLA-E*01:03 (Fig. 1B). Similarly, VLAPRTLLL variants with Gly at position 8 and Ala at position 9 could not bind to HLA-E*01:03 as well (Fig. 1B). The VMAPRTLIL variant with Asp at position 7 was the only variant that could not bind to HLA-E*01:03 (Fig. 1A). Most of the peptide variants had an affinity comparable to the wildtype VL9-peptides (Fig. 1A and B). Affinity of the variants with substitutions at position 6, 7, and 9 strongly depended on the residue. For instance, variants with Pro, a residue that significantly constraints the peptide conformation, at position 6 and 7 of VMAPRTLIL and VLAPRTLLL were strong binders, whereas variants with charged residues at these positions had a decreased binding affinity. In addition, the position 9 variant with Phe was a very strong binder, whereas variants with Tyr and Trp were moderate binders (Fig. 1A and B).

VMAPRTLIL and VLAPRTLLL peptides have a high affinity for HLA-E*01:03. In previous studies, we have identified multiple HLA-E restricted Mtb-derived peptides that generally have a low affinity for HLA-E*01:03.^4^^,^^31^ We included 3 Mtb-derived peptides, named Mtb34, 44 and 55, in the current analysis to investigate if substituting the central part of these peptides (positions 3 to 6) with the residues present in VL9-peptides (ie, APRT) affects the binding affinity for HLA-E*01:03, including recognition by TCRs and NKG2A/CD94. Substituting Mtb34 and Mtb55 with the central residues of VL9 increased the affinity for HLA-E*01:03 relative to the wildtype peptides (Fig. 1C). In contrast, Mtb44-VL9 hybrid variants had a binding affinity comparable to Mtb44 (Fig. 1C).

Thus, although residue dependent, the majority of peptide variants could bind to HLA-E*01:03.

### Peptide positions 4 to 9 are critical for HLA-E/peptide interactions with both TCR and NKG2A/CD94

**Figure 2.**
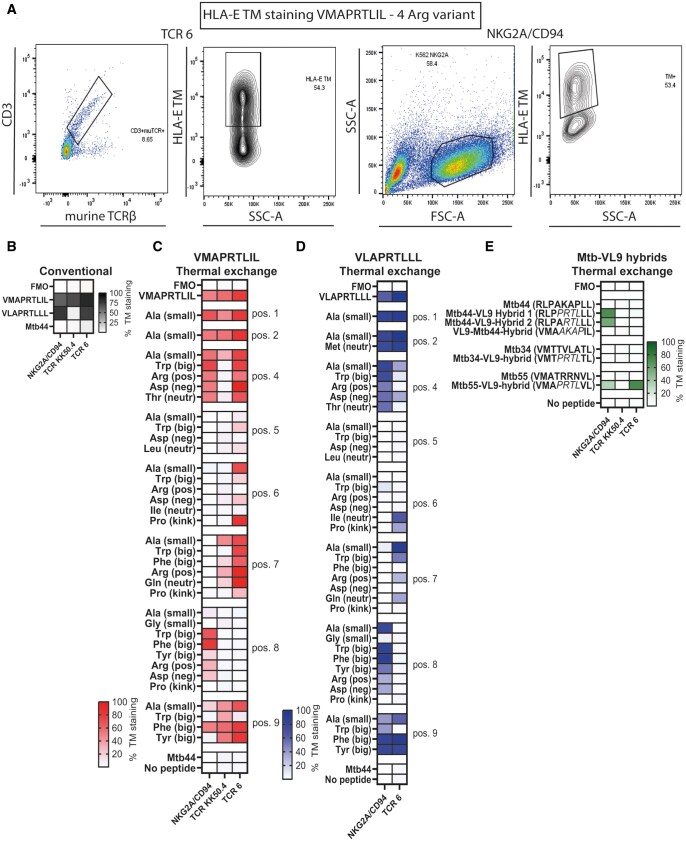
NKG2A/CD94 and TCRs have different requirements for interacting with the HLA-E/peptide complex, localized to positions 4 to 9. K562 cells expressing the NKG2A/CD94 co-receptor (K562-NKG2A/CD94) and Jurkat cells transduced with TCR KK50.4 (recognizing the sequence VMAPRTLIL) and TCR 6 (recognizing VL9 peptides) were stained with HLA-E*01:03 TMs. (A) Representative density plot showing staining with the VMAPRTLIL-Arg 4 variant on Jurkat TCR 6 cells (left) and K562-NKG2A/CD94 cells (right). Jurkat TCR 6 and KK50.4 cells were stained for CD3 and murine TCR-β to gate on the TCR expressing population. (B) Heatmap showing the percentage conventional HLA-E TM staining for the wildtype sequences VMAPRTLIL, VLAPRTLLL and Mtb44 on K562-NKG2A/CD94, Jurkat TCR KK50.4 and TCR 6 cells. (C) Heatmap showing the percentage thermal exchanged HLA-E TM staining on K562-NKG2A/CD94, Jurkat TCR KK50.4 and TCR 6 cells with the VMAPRTLIL variants. (D) Same as C, but then for the VLAPRTLLL variants. (E) Same as C, but then for the Mtb-VL9 hybrid peptides. Residues that were changed in the hybrid peptides relative to the wildtype peptides are shown in italics. Thermal exchanged HLA-E TMs were tested once for binding to NKG2A/CD94, TCR 6 and TCR KK50.4.

The peptide variants were folded into HLA-E TMs using the temperature exchange method to determine the differences in HLA-E/peptide interactions between TCR KK50.4 [specific for VMAPRTLIL], TCR 6 [specific for both VL9-peptides] and NKG2A/CD94 (Fig. 2A for staining examples). HLA-E TM staining on TCR transduced cells was only evaluated on the TCR expressing population (Fig. 2A). TCR 6 was identified in our previous study via sorting CD8^+^ T cells recognizing HLA-E*01:03 TMs folded with VL9-peptides and had a clonal count of 105/298.^23^ The temperature exchanged HLA-E TMs could bind to LILRB1 indicating correct monomer folding and tetramerization (Fig. S2). Additionally, conventional TMs folded with Mtb44, VMAPRTLIL and VLAPRTLLL were included as positive HLA-E TM controls and to verify the peptide specificity of the TCRs and NKG2A/CD94 (Fig. 2B). VMAPRTLIL and VLAPRTLLL peptides in complex with HLA-E can interact with both NKG2A/CD94 and TCR 6, whereas KK50.4 could only interact with the HLA-E/VMAPRTLIL complex, confirming specificity for VMAPRTLIL (Fig. 2B).^9^ Therefore, VLAPRTLLL variants were not evaluated for TCR KK50.4.

Critical positions for receptor interaction with the HLA-E/peptide complex were determined using VMAPRTLIL and VLAPRTLLL variants containing Ala substitutions at each residue position, except for position 3, which contains Ala in the wildtype sequence. The Ala scanning revealed that positions 5 to 9 were critical for interaction with NKG2A/CD94 and TCR 6 based on the reduced binding of HLA-E TMs folded with these Ala variants. In contrast, positions 4 to 9 were critical for interaction with TCR KK50.4 (Fig. 2C and D). As the Ala positions 1 to 3 variants were recognized by both TCRs and NKG2A/CD94, these positions are most likely not involved in receptor interaction (Fig. 2C and D). Therefore, variants with residues introducing a different size or charge at positions 4 to 9 were used to further characterize receptor recognition of HLA-E/peptide complexes (Fig. S1).

The substitutions at positions 5 to 7 diminished the interaction with NKG2A/CD94, whereas all substitutions at position 4 and most substitutions at positions 8 and 9 were tolerated (Fig. 2C and D). For both TCRs, substitutions at position 5 and 8 diminished receptor interaction, whereas most substitutions at positions 4, 6, 7, and 9 were tolerated (Fig. 2C and D). Moreover, peptide affinity (Fig. 1) was not correlated to HLA-E TM staining, thus peptide binding to HLA-E does not directly translate to receptor recognition (Fig. S4A–C). Similarly, HLA-E TM staining between TCR and NKG2A/CD94 expressing cells was not correlated suggesting that these receptors interact in a different manner with the HLA-E/peptide complex (Fig. S5A, B for correlations). Especially substitutions at position 7 and 8 had opposite effects. Substitutions at position 7 abrogated interaction with NKG2A/CD94, demonstrating importance for interaction with NKG2A/CD94, whereas substitutions at position 8 abrogated interaction with TCR KK50.4 and 6, demonstrating importance for interaction with the TCRs. HLA-E in complex with position 5 variants did not interact with TCR KK50.4 and NKG2A/CD94, and interacted to a limited extent with TCR 6. These findings suggest that Arg in the wildtype peptide is crucial for interaction with NKG2A/CD94 and TCRs. In addition, NKG2A/CD94 and TCR KK50.4 and 6 tolerated small and hydrophobic/bulky residues at position 9, suggesting flexibility at this position for interaction with both receptors. Flexibility was also observed at positions 4 and 8 for NKG2A/CD94 and positions 4 and 7 for the 2 TCRs. Thus, the interaction with the HLA-E/peptide complex at positions 4, 6, 7, 8, and 9 was not restricted to the wildtype residue.

HLA-E TM staining was only moderately correlated between TCR KK50.4 and TCR 6 (R^2^ = 0.35), indicating that these TCRs have different interaction requirements (Fig. 2C and D, and Fig. S5C). VMAPRTLIL variants with hydrophobic, bulky and charged residues at position 4 interacted with TCR 6, whereas TCR KK50.4 only interacted with the Ala variant. None of the VMAPRTLIL variants at position 6 could interact with TCR KK50.4, whereas Pro and Ala variants interacted with TCR 6. In addition, VMAPRTLIL variants with hydrophobic/bulky, small and charged residues at position 7 interacted with TCR 6, whereas variants with small and charged residues interacted with TCR KK50.4. Thus, the difference between TCR KK50.4 and 6 was localized to positions 4, 6, and 7 and reveal that the HLA-E/peptide interaction with TCR KK50.4 is likely more restricted than with TCR 6.

Consequently, the interaction of HLA-E/peptide complexes with NKG2A/CD94 and TCRs was mainly localized to the central part of the peptide (ie, positions 4 to 6). We therefore studied if Mtb peptides substituted with the central residues of VL9-peptides (Mtb-VL9 hybrids) can interact with NKG2A/CD94 or TCRs, as the wildtype Mtb peptides cannot (Fig. 2E). HLA-E in complex with the Mtb44-VL9 hybrids and the Mtb55-VL9 hybrid could interact with NKG2A/CD94, while the wildtype HLA-E/Mtb complexes could not (Fig. 2E). In contrast, TCR KK50.4 could not interact with any of the wildtype or hybrid peptides in complex with HLA-E, whereas TCR 6 could interact with the Mtb55-VL9 hybrid (Fig. 2E). These results suggest that the interaction of NKG2A/CD94 with the HLA-E/peptide complex is presumably more flexible than for TCRs and possibly relies more on the conformation of the HLA-E/peptide complex.

### HLA-E/peptide complexes that can interact with NKG2A/CD94 or TCRs can also induce receptor signaling

**Figure 3.**
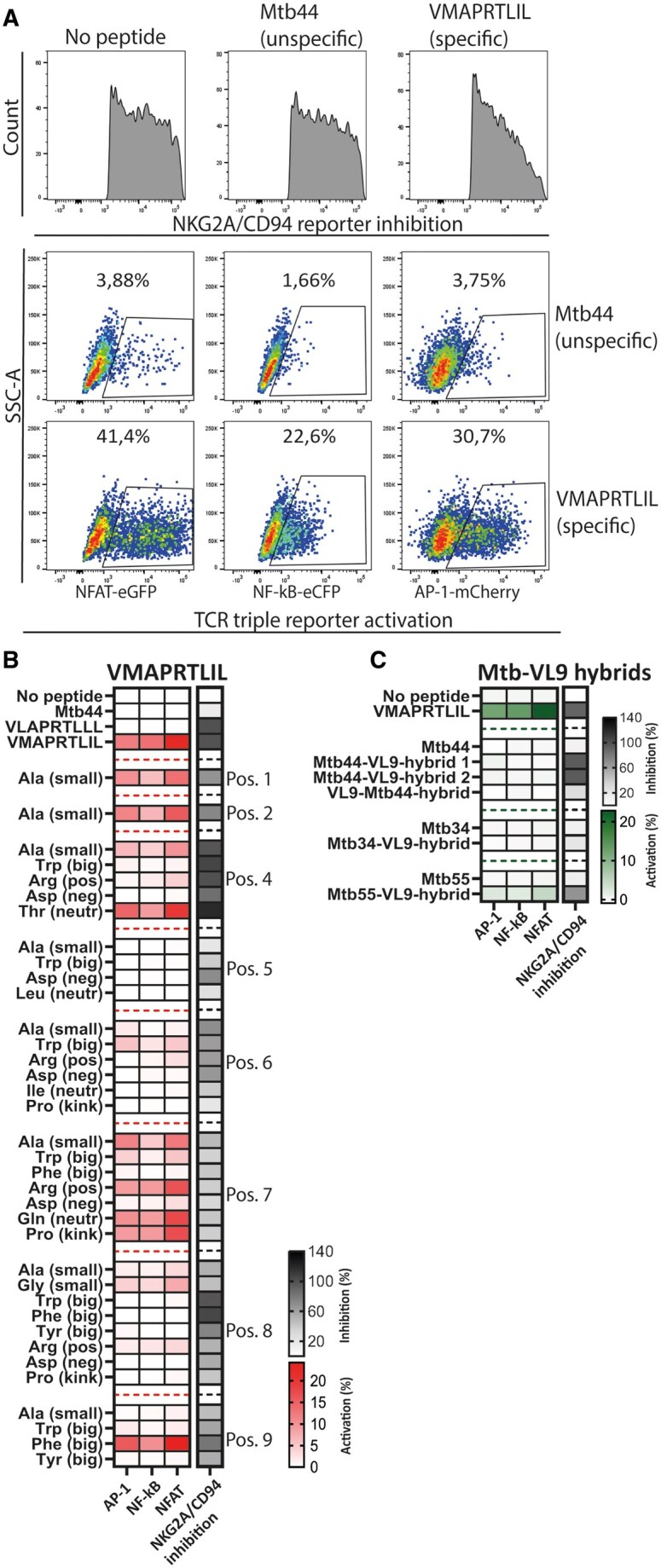
HLA-E induced signaling of TCR and NKG2A/CD94 depends on the peptide sequence. JE6.1 NKG2A/CD94 NF-kB:eGFP reporter cells and Jurkat TCR KK50.4 triple reporter cells were stimulated with K562 HLA-E*01:03 cells presenting VMAPRTLIL and Mtb-VL9 hybrid peptides. (A) Upper panel: representative plots showing the reporter inhibition signal (eGFP) after stimulating JE6.1 NKG2A/CD94 cells with K562 HLA-E*01:03 cells unloaded with peptide or loaded with Mtb44 (unspecific) or VMAPRTLIL (specific). Reduction of the reporter signal upon peptide stimulation relative to stimulation with unloaded HLA-E*01:03 cells was used to determine the inhibitory signal of the peptide. Lower panel: representative plots showing activation of the triple reporters NFAT (eGFP), AP-1 (mCherry) and NF-kB (eCFP) after stimulating Jurkat TCR KK50.4 cells with K562 HLA-E*01:03 cells loaded with Mtb44 (unspecific) or VMAPRTLIL (specific). (B) Heatmap showing the percentage activation of the TCR triple reporters and percentage inhibition of the NKG2A/CD94 reporter after stimulation with the VMAPRTLIL peptide variants. (C) Same as B, but then for Mtb-VL9 hybrids. Values represent the median percentage reporter activation or inhibition (n = 3 with 2 technical replicates for TCR KK50.4 and n = 2 with 2 technical replicates for NKG2A/CD94).

The capacity of the peptide variants to induce receptor signaling was determined using reporter cell lines expressing NKG2A/CD94 or TCR KK50.4. Activation of TCR KK50.4 was based on signaling of the NFAT, NF-kB, and AP-1 triple reporters and activation of NKG2A/CD94 was based on the inhibition of a single reporter (Fig. S3B for schematic overview of the NKG2A/CD94 reporter system). HLA-E expressing cells were loaded with the peptide variants to determine the percentage activation of the TCR KK50.4 triple reporters and the percentage inhibition of the NKG2A/CD94 reporter relative to the positive control (VMAPRTLIL) and negative controls (no peptide and Mtb44) (Fig. 3A).

The percentage reporter inhibition or activation for NKG2A/CD94 and TCR KK50.4, respectively, was determined for the VMAPRTLIL variants and the Mtb wildtype or hybrid peptides (Fig. 3B and C). The NKG2A/CD94 reporter inhibition data showed results similar to HLA-E TM staining (Fig. 3B), though with less clarity due to background fluorescence (R^2^ = 0.62, Fig. S5D). The TCR KK50.4 triple reporters show consistent activation for each peptide with the highest activation for NFAT and the lowest for AP-1, which likely results from different kinetics between these transcription factors (Fig. 3B). HLA-E TM staining and TCR KK50.4 triple reporter activation were weakly correlated (R^2^ = 0.39, Fig. S5E) with variants like Pro at position 7, Thr at position 4, and Ala and Tyr at position 9 showing opposite effects on binding and signaling (Fig. S5E). Similar to HLA-E TM binding, substitutions at positions 5, 6, and 8 diminished TCR KK50.4 triple reporter activation, suggesting importance for binding and signaling. In contrast, substitutions at 4, 7, and to a lesser extent 9 (only the Phe variant) still induced TCR KK50.4 activation, suggesting that these residues are less critical for TCR interaction and signaling.

The most notable difference between TCR KK50.4 and NKG2A/CD94 reporter signaling was located to positions 7 and 8 of the peptide; position 7 was more involved in NKG2A/CD94 signaling, while position 8 was more critical for TCR KK50.4 signaling, consistent with the HLA-E TM staining data (Fig. 2B). Position 5 and 6 were crucial for signaling by both receptors, also in line with HLA-E TM staining, as these variants induced limited TCR KK50.4 activation or NKG2A/CD94 inhibition. The Mtb44 and Mtb55-VL9 hybrids can induce signaling by NKG2A/CD94 but hardly by TCR KK50.4, further substantiating that NKG2A/CD94’s interaction with HLA-E/peptide complexes is likely more flexible than for TCRs (Fig. 3C).

### Selectivity for position 8 for interaction with the TCRs and position 7 for interaction with NKG2A/CD94

**Figure 4.**
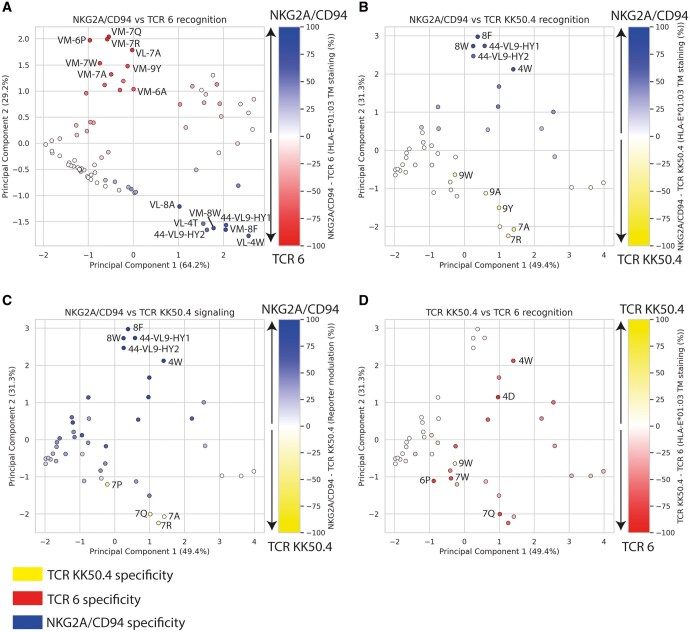
NKG2A/CD94 and TCRs have different recognition and signaling requirements for the HLA-E/peptide complex. (A) Principal component analysis (PCA) on the percentage HLA-E TM staining of the VMAPRTLIL (VM) and VLAPRTLLL (VL) variants on TCR 6 and NKG2A/CD94 expressing cells. Dots are colored based on specificity for either NKG2A/CD94 or TCR 6, shown in the legend. (B) Similar as A, but then between TCR KK50.4 and NKG2A/CD94 expressing cells for the VMAPRTLIL variants. (C) PCA on the reporter signals of TCR KK50.4 and NKG2A/CD94 expressing cells for the VMAPRTLIL variants. (D) Similar as A, but then between TCR KK50.4 and TCR 6 expressing cells for the VMAPRTLIL variants. The top 10% peptides selective for each receptor are named using the single letter amino acid abbreviation, except for (D) as one peptide was recognized by TCR KK50.4. To differentiate between substitutions in the VMAPRTLIL or VLAPRTLLL peptides, the peptide abbreviations in (A). include VM or VL. Substitutions in (B–D) are only in VMAPRTLIL and codes thus refer to peptide position and substitution.

Principal component analysis (PCA) was performed on the HLA-E TM staining and reporter activation data to identify the most selective peptides for interaction with either NKG2A/CD94 or TCR KK50.4 and 6 (Fig. 4A–D). Loadings for the PCA’s can be found in Fig. S6. HLA-E TM staining with the VMAPRTLIL and VLAPRTLLL variants showed clear separation between NKG2A/CD94 and TCR 6 (Fig. 4A). Position 7 variants were selective peptides for interaction with TCR 6, especially variants with small and charged residues (i.e., Gln, Ala and Arg), followed by position 6 (Pro and Ala) and 9 (Tyr). Position 8 variants with small and larger residues (Ala, Phe, and Trp) were selective peptides for interaction with NKG2A/CD94, followed by position 4. These results further confirm that variants at position 7 and 8 have opposite effects on the interaction with NKG2A/CD94 and TCR 6. Although separation based on HLA-E TM staining was less clear between TCR KK50.4 and NKG2A/CD94, position 7 VMAPRTLIL variants were the most selective peptides for interaction with TCR KK50.4 and position 8 variants for NKG2A/CD94 (Fig. 4B), similar to the comparison between TCR 6 and NKG2A/CD94. Separation of VMAPRTLIL peptides based on reporter signaling between NKG2A/CD94 and TCR KK50.4 revealed that more peptide variants selectively activated NKG2A/CD94 relative to TCR KK50.4. This was mainly because of the background signal of the NKG2A/CD94 reporter skewing these peptides as more selective for NKG2A/CD94 (Fig. 4C). Also in this comparison, position 7 variants induced the highest activation of TCR KK50.4 triple reporters and position 8 variants the highest inhibition of the NKG2A/CD94 reporter.

The interaction of the HLA-E/peptide complex with TCR KK50.4 was more restricted to the wildtype residues compared to TCR 6 thereby hardly permitting variants to interact (Fig. 4D). The position 9 variant with Trp was the only peptide variant that was recognized by TCR KK50.4 and not TCR 6. Hence, recognition of HLA-E/peptides by TCR KK50.4 and TCR 6 involved different residues, possibly because of the distinct TCR-αβ composition of both TCRs.

## Discussion and conclusion

TCRs and NKG2A/CD94 are 2 receptor families that can interact with the HLA-E/peptide complex. In this study, we directly compared 2 HLA-E restricted TCRs and NKG2A/CD94 for interaction and signaling requirements using VL9-derived peptide variants. Positions 5 and 6 were involved in interaction with NKG2A/CD94 and the 2 TCRs, whereas positions 2, 3, 4, and to some extent also position 9, were not essential for receptor interaction. Positions 2 and 9 are the primary anchor residues for peptide binding to HLA-E and are therefore possibly unavailable to directly interact with the receptors.^7^^,^^15^ In agreement with our results, position 5 and 6 were identified before as the main contact residues with NKG2A/CD94 via non-covalent interactions with the Gln at position 112 of CD94.^21^ For TCR KK50.4, it was shown before that positions 5, and to a lesser extent position 6, directly interact with the TCR via the CDR3-α and -β loops.^25^

Position 8 was the most critical position for TCR interaction with the HLA-E/peptide complex and position 7 for NKG2A/CD94, based on the absence of interactions and signaling with peptide variants for these positions. Previously, it was shown that position 7 contributes indirectly to HLA-E NKG2A/CD94 interactions as it facilitates positioning of adjacent residues to interact with NKG2A/CD94.^20^ Crystal structures and structural models are available for NKG2A/CD94 in complex with HLA-E presenting the VL9-peptide VMAPRTLFL.^21^^,^^32^ These models showed that the CD94 subunit makes more contacts with the peptide than NKG2A, accounting for 80% of the interactions compared to 20% for NKG2A.^32^ The Gln at position 112 of CD94 is the main residue that interacts with peptide positions 5, 6 and 8 via hydrogen bonding or electrostatic interactions. These positions were also identified as contact residues in this study.

We also observed that the interaction of NKG2A/CD94 with the HLA-E/peptide complex was dependent on the residue at position 8. For instance, variants with large and hydrophobic residues, such as Phe, at position 8 were best recognized by NKG2A/CD94, whereas all other residues, including small and charged ones, were limitedly recognized. In line with our findings, the peptide sequence VMAPRTLFL, which is identical to the HLA-G-derived VL9-peptide, induced the highest level of NKG2A/CD94 signaling among other VL9-peptides previously.^20^ Additionally, another study showed that primary NKG2C^+^ NK cells induced the highest level of CD107a expression and cytokine secretion after stimulation with HLA-E/VMAPRTLFL presenting cells compared to other position 8 variants derived from clinical CMV isolates.^33^ Collectively, these findings illustrate that induction of NK cell responses mediated by NKG2A or -C/CD94 strongly depends on the residue at position 8.

Published crystal structures of TCR KK50.4 in complex with HLA-E presenting the peptide VMAPRTLIL revealed that the TCR-α and -β chain interact with the peptide to an equal extent. Specifically, the Ile at position 8 makes direct interactions with the CDR1, 2, and 3-β loops, whereas both CDR3-α and -β loops interact with the Arg and Pro at positions 5 and 6, respectively.^25^^,^^32^ The Leu at position 7 does not interact with TCR KK50.4. These findings based on the crystal structure of TCR KK50.4 are in agreement with our findings.^25^ Although TCR KK50.4 is well-characterized, we show in our study that positions 5, 6, and 8 are also critical for the interaction with a newly described HLA-E/VL9 restricted TCR, named TCR 6.^25^

TCR recognition depends on the conformation of the peptide in the peptide binding groove of HLA-E.^34^ For instance, a Mtb-derived high affinity HLA-E restricted peptide called Mtb44 (RLPAKAPLL) has a completely different sequence compared to VL9-peptides but adopts an identical conformation in the peptide binding groove as VL9-peptides.^34^ In addition, the Mtb44-restricted TCR docks in a mode similar to TCR KK50.4, facilitating direct interactions with the peptide.^34^ In contrast, a TCR recognizing a low affinity HIV-derived peptide that adopts a divergent conformation in the HLA-E peptide binding groove, docks in a non-canonical mode. This TCR makes broader interactions with the HLA-E/peptide complex rather than restricted to single residues.^34^ Next to the peptide conformation, HLA-E in complex with lower affinity peptides forms heterogenous structures, whereas rigid structures form when bound to high affinity peptides.^24^ Interactions between the HLA-E/peptide complex and TCRs or NKG2A/CD94 is therefore likely also mediated by the conformation of the resulting HLA-E/peptide complex. The variants in our study might induce a particular conformation of the HLA-E/peptide complex facilitating interaction with these receptors. Possibly, the HLA-E/peptide complex conformation is more relevant for NKG2A/CD94 interaction/signaling than for TCRs as the Mtb-VL9 hybrid peptides, which presumably adopt a similar conformation in the peptide binding groove as VL9-peptides given their high affinity for HLA-E*01:03, can interact with NKG2A/CD94 and not with the TCRs. The Mtb-VL9 hybrid peptides contain substantial changes compared to the VL9-peptides. HLA-E presenting these peptides could not be recognized by TCR KK50.4 and TCR 6. Although other TCRs might be more flexible in the recognition of HLA-E/peptide complexes, our findings illustrate that TCR KK50.4 and TCR 6 recognize HLA-E/peptide complexes with high specificity.

We observed that the interaction of TCR KK50.4 with the HLA-E/peptide complex is more restricted than for TCR 6. TCR 6 has also greater flexibility as it can recognize both the wildtype VMAPRTLIL and VLAPRTLLL peptides. This might be caused by the different variable TCR-α and TCR-β composition of these TCRs resulting in a different docking mode of the TCRs onto the HLA-E/peptide complex.

We only evaluated interaction and signaling requirements for NKG2A/CD94 but HLA-E/VL9 complexes can also interact with NKG2C/CD94. These two receptors have opposing functions; NKG2A/CD94 inhibits NK cell-mediated lysis, whereas NKG2C/CD94 activates lysis upon recognition of HLA-E/VL9 complexes. Both receptors can be co-expressed on NK cells and thus NK cells must process input from both receptors before exerting a functional response. However, NKG2A/CD94 has a higher affinity for HLA-E/peptide complexes than NKG2C/CD94. It was also found that NKG2A/CD94 induces a stronger inhibitory signal compared to the activation signal induced by NKG2C/CD94 upon recognition of identical HLA-E/peptide complexes.^18^^,^^20^ Nevertheless, NKG2A/CD94 and NKG2C/CD94 have a comparable recognition motif for HLA-E, as determined previously using peptide libraries,^18^ and we expect that our results for NKG2A/CD94 will be similar for NKG2C/CD94.

Receptor interaction and signaling for the HLA-E/peptide complex was correlated, especially for NKG2A/CD94, which indicates that interaction with the receptor is required to induce signaling. HLA-E/peptide binding affinity and receptor interaction were not correlated demonstrating that high affinity peptides do not necessarily interact with the receptor. Our findings may also help to select HLA-E restricted peptides that are either recognized by the TCR or by NKG2A/CD94. For instance, selection of HLA-E/peptides that are recognized by TCRs and not by NKG2A/CD94 may act as dual edged sword vaccine components, exerting both T cell and NK cell cytolytic activity against the infected cell.

Our study was limited by the number of HLA-E specific TCRs included and should be extended to know if our findings can be translated to other TCRs recognizing HLA-E/peptides. Furthermore, we evaluated differences between NKG2A/CD94 and TCRs based on interaction and signaling of peptide variants, but molecular studies are needed to identify the specific non-covalent interactions between both receptors and the HLA-E/peptide complex. Lastly, our findings were limited by the number of peptide modifications included.

In conclusion, we show that NKG2A/CD94 and TCRs differentially interact with HLA-E/peptide complexes.

## Supplementary Material

vkae068_Supplementary_Data

## Data Availability

The data underlying this article are available in the article and in its online supplementary material.
