# Supplementary material for: HLA-E/peptide complexes differentially interact with NKG2A/CD94 and T cell receptors
Source: J Immunol. 2025 Mar 14;214(4):595–605. doi: 10.1093/jimmun/vkae068 (PMC12041767; doi:10.1093/jimmun/vkae068)
Supplement: vkae068_Supplementary_Data [file vkae068_supplementary_data.zip › vkae068_Supplementary_Data/JIMMUN-24-00593-s01.docx]

## Supplemental Data

|  | 1 | 2 | 3 | 4 | 5 | 6 | 7 | 8 | 9 |
| --- | --- | --- | --- | --- | --- | --- | --- | --- | --- |
|  | **V** | **M/L** | **A** | **P** | **R** | **T** | **L** | **I/L** | **L** |
| Substitutions | Ala | Ala | Ala | Ala | Ala | Ala | Ala | Ala | Ala |
|  |  | Met |  | Trp | Trp | Trp | Trp | Gly | Trp |
|  |  |  |  | Arg | Asp | Arg | Phe | Trp | Phe |
|  |  |  |  | Asp | Leu | Asp | Arg | Phe | Tyr |
|  |  |  |  | Thr |  | Ile | Asp | Tyr |  |
|  |  |  |  |  |  | Pro | Gln | Arg |  |
|  |  |  |  |  |  |  | Pro | Asp |  |
|  |  |  |  |  |  |  |  | Pro |  |

**Supplementary Figure 1. Overview of the VMAPRTLIL and VLAPRTLLL variants included in the analysis.** Substitutions at each position are represented with the triple letter abbreviation for amino acids.


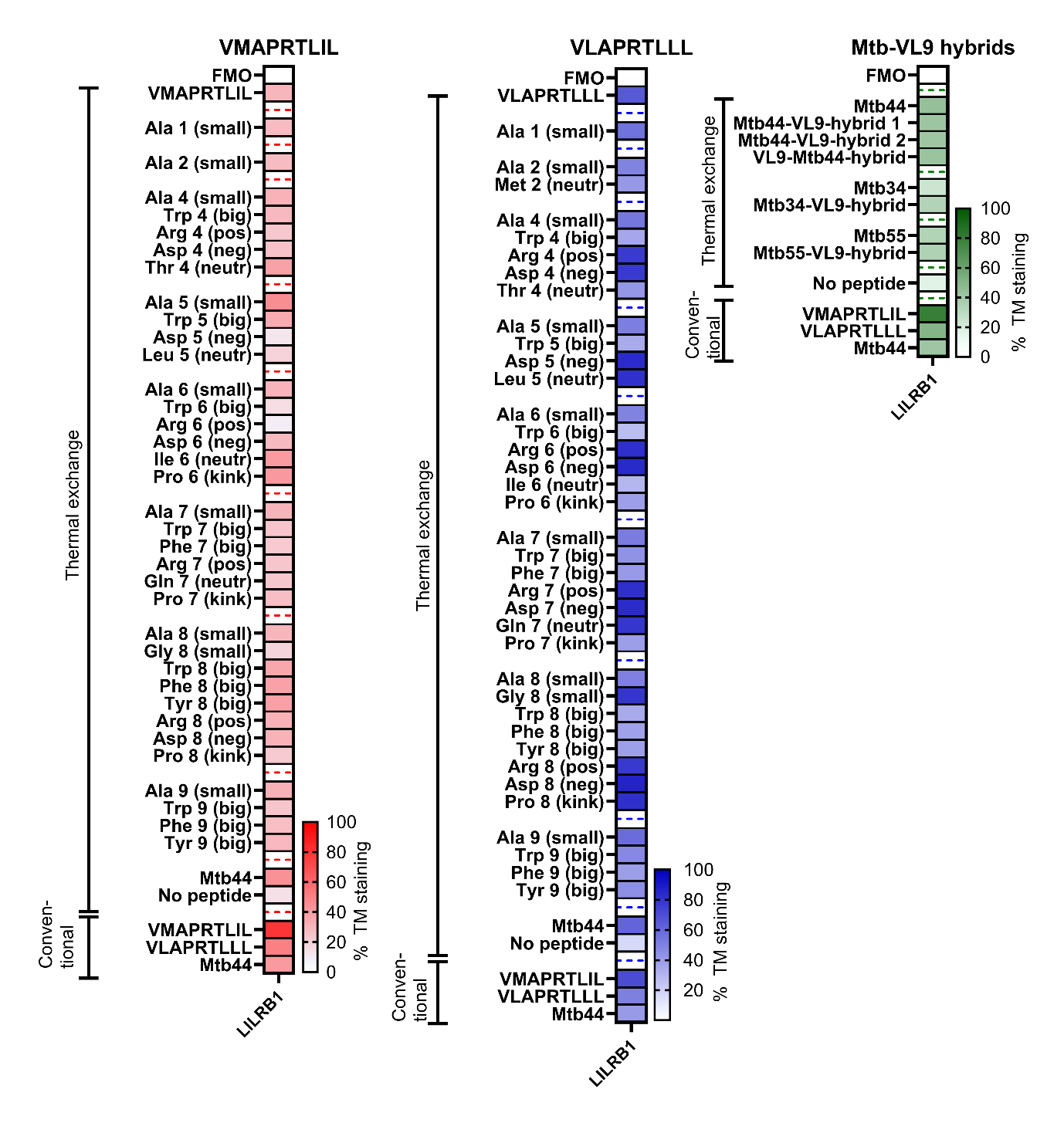
**Supplementary Figure 2. HLA-E*01:03 TM staining on LILRB1 expressing cells.** Heatmaps show the percentage staining with thermal and conventional HLA-E TMs folded with the VMAPRTLIL variants (left), VLAPRTLLL variants (middle) and Mtb-VL9 hybrids (right) on K562 LILRB1 expressing cells.


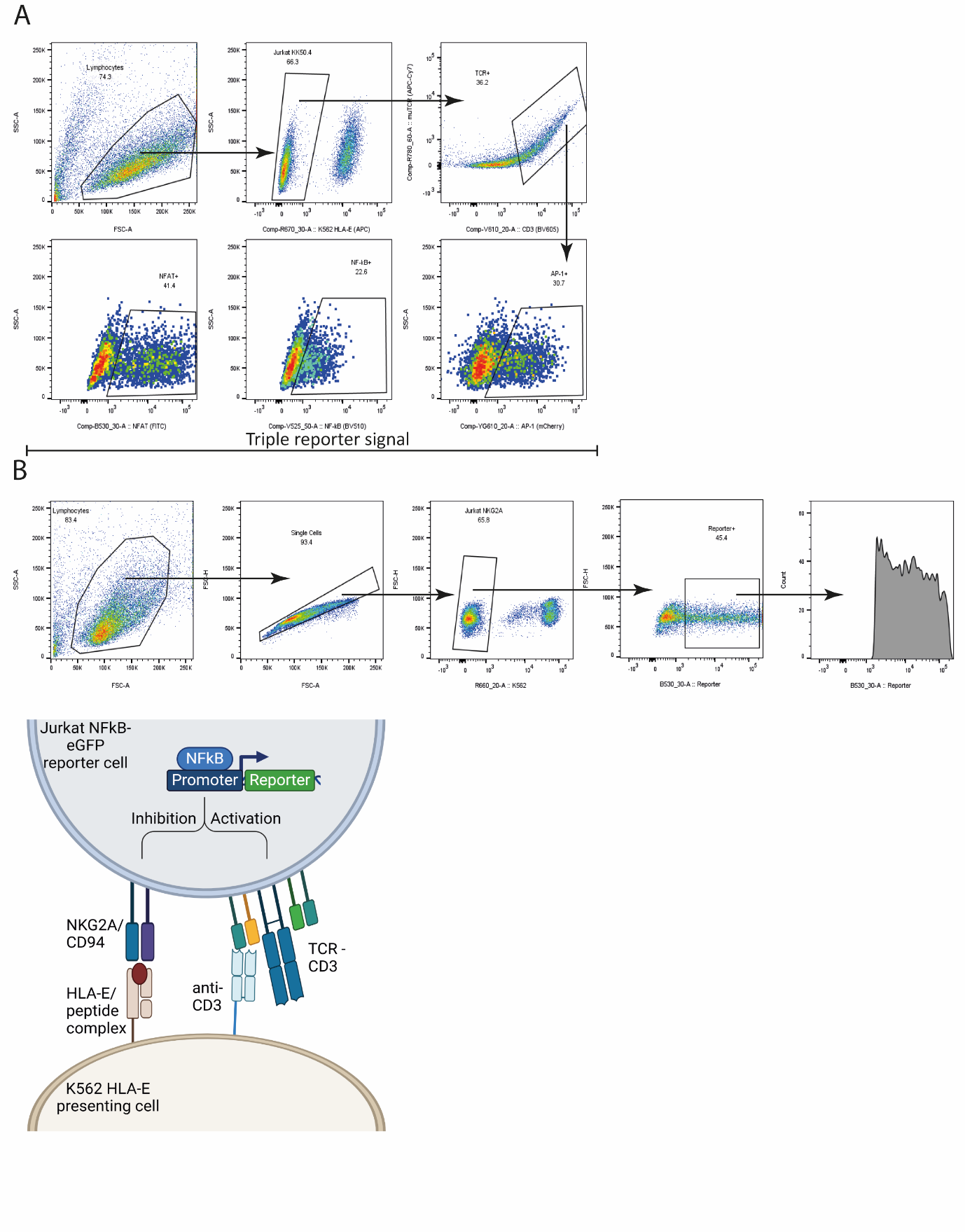


**Supplementary Figure 3. Gating strategies to determine TCR reporter activation and NKG2A/CD94 reporter inhibition. A.** Gating strategy to determine the percentage triple reporter activation in TCR-expressing JE6.1 CD8^+^ cells after stimulation with peptide-loaded K562 HLA-E*01:03 cells. This comprised the subsequent gating on the total population (1), the Jurkat specific population (APC-neg, excluding K562 cells) (2), TCR expressing population based on muTCR and CD3 staining (3) and the positive reporter signals (4). **B.** Gating strategy to determine NKG2A/CD94 reporter inhibition after stimulation with peptide-loaded K562 HLA-E*01:03 cells. This comprised the subsequent gating on the total population (1), single cells (2), Jurkat NKG2A/CD94 specific population (APC-neg, excluding K562 cells) (3) and reporter positive population (4). The cartoon shows a schematic representation of the NKG2A/CD94 reporter inhibition assay as is also published by Battin et al. 2022(29). The reporter signal is activated via the interaction between TCR/CD3 expressed on Jurkat cells and anti-CD3 expressed on K562 cells. This signal can be reduced if the HLA-E/peptide complex expressed on K562 cells is recognized by NKG2A/CD94 expressed on Jurkat cells. The combined reporter signal is subsequently measured using Flow cytometry. Cartoon is created in Biorender.com.

**
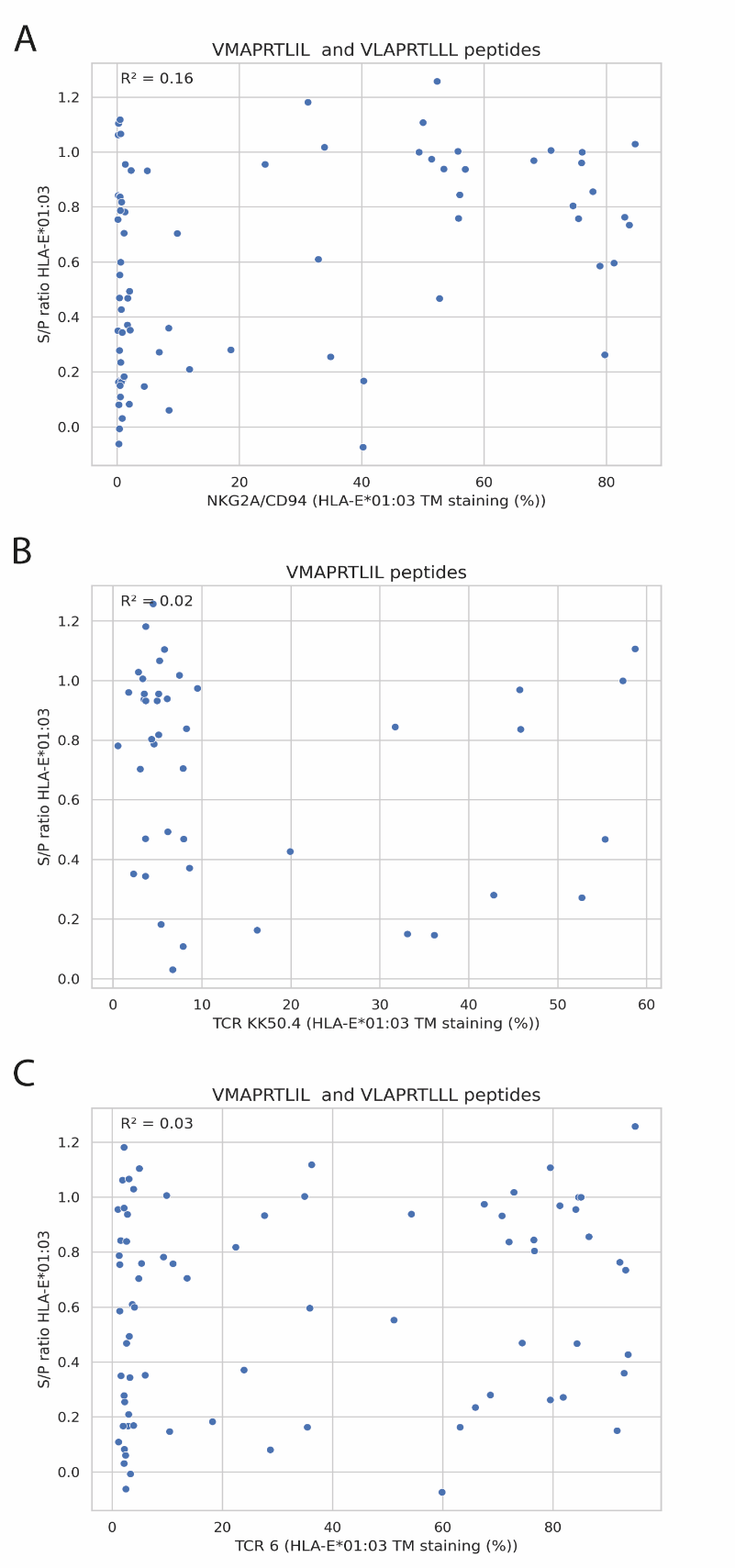
**

**Supplementary Figure 4. Correlation plots between HLA-E*01:03 peptide affinity and HLA-E TM recognition. A.** Correlation between HLA-E TM staining on NKG2A/CD94 expressing cells (X-axis) and HLA-E*01:03 peptide affinity (Y-axis). This correlation includes all VMAPRTLIL and VLAPRTLLL variants. **B.** Correlation between HLA-E TM staining on TCR KK50.4 expressing cells (X-axis) and HLA-E*01:03 peptide affinity (Y-axis). This correlation includes all VMAPRTLIL variants. **C.** Correlation between HLA-E TM staining on TCR 6 expressing cells (X-axis) and HLA-E*01:03 peptide affinity (Y-axis). This correlation includes all VMAPRTLIL and VLAPRTLLL variants.

**
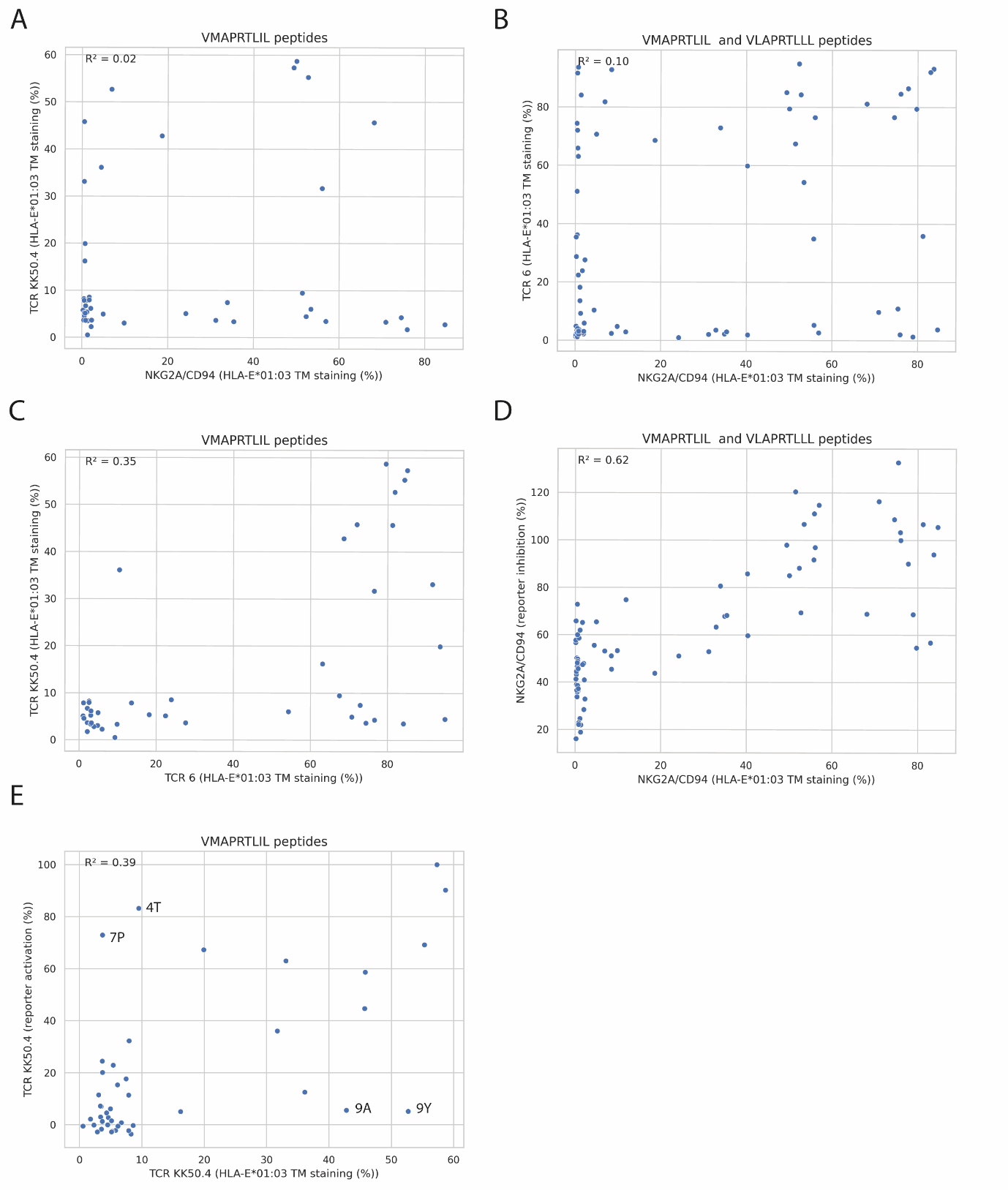
**

**Supplementary Figure 5. Correlation plots between TCRs and NKG2A/CD94 for HLA-E TM staining and reporter signaling. A.** Correlation plot between NKG2A/CD94 and TCR KK50.4 for HLA-E TM staining with the VMAPRTLIL variants. **B.** Correlation plot between NKG2A/CD94 and TCR 6 for HLA-E TM staining with the VMAPRTLIL and VLAPRTLLL variants. **C.** Correlation plot between TCR KK50.4 and TCR 6 for HLA-E TM staining with the VMAPRTLIL variants. **D.** Correlation plot between HLA-E TM staining and inhibitory signaling of NKG2A/CD94 expressing cells for the VMAPRTLIL and VLAPRTLLL variants**. E.** Correlation plot between HLA-E TM staining and activation of the NFAT reporter of TCR KK50.4 expressing cells for the VMAPRTLIL variants.


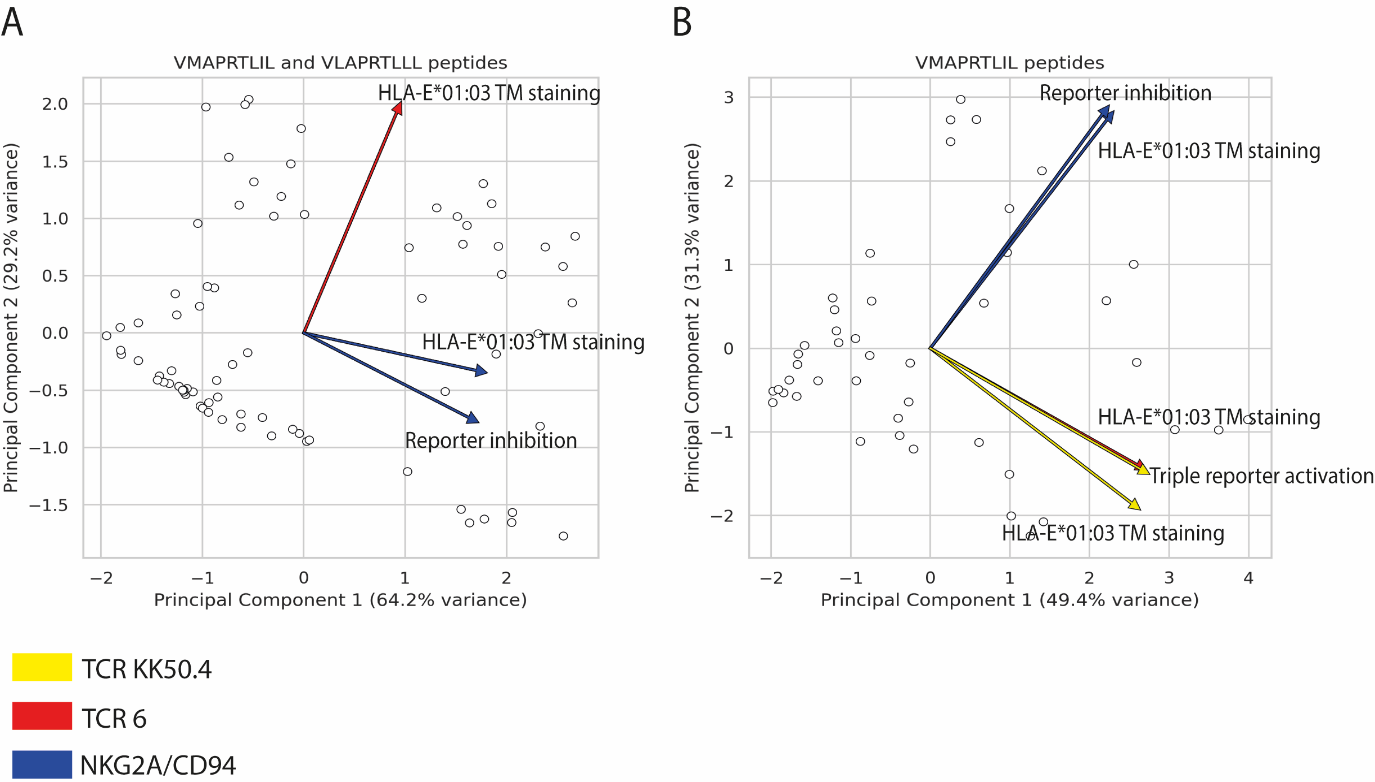


**Supplementary Figure 6. PCA loadings for the PCA’s shown in Figure 4.** PCA loadings describe how much (arrow length) the peptides contributed to the PCA and in which direction (arrow direction). **A.** PCA loadings for the VMAPRTLIL and VLAPRTLLL peptides for TCR 6 (red arch) and NKG2A/CD94 (blue arrow). **B.** PCA loadings for VMAPRTLIL peptides for TCR 6 (red arrow), NKG2A/CD94 (blue arrow) and TCR KK50.4 (yellow arrow).

**Supplementary Table 1. Amino acid composition of the peptide variants.** Table is categorized for VMAPRTLIL variants (left), VLAPRTLLL variants (middle) and Mtb-VL9 hybrid peptides (right).

| **VMAPRTLIL variants** | | **VLAPRTLLL variants** | | **Mtb-VL9 hybrids** | |
| --- | --- | --- | --- | --- | --- |
| **Sequence** | **Abbreviation** | **Sequence** | **Abbreviation** | **Sequence** | **Abbreviation** |
| AMAPRTLIL | Ala 1 (small) | ALAPRTLLL | Ala 1 (small) | RLPAKAPLL | Mtb44 |
| VAAPRTLIL | Ala 2 (small) | VAAPRTLLL | Ala 2 (small) | RLPPRTLLL | Mtb44-VL9-hybrid 1 |
| VMAARTLIL | Ala 4 (small) | VMAPRTLLL | Met 2 (neutr) | RLPARTLLL | Mtb44-VL9-hybrid 2 |
| VMAWRTLIL | Trp 4 (big) | VLAARTLLL | Ala 4 (small) | VMAAKAPIL | VL9-Mtb44-hybrid |
| VMARRTLIL | Arg 4 (pos) | VLAWRTLLL | Trp 4 (big) | VMTTVLATL | Mtb34 |
| VMADRTLIL | Asp 4 (neg) | VLARRTLLL | Arg 4 (pos) | VMTPRTLTL | Mtb34-VL9-hybrid |
| VMATRTLIL | Thr 4 (neutr) | VLADRTLLL | Asp 4 (neg) | VMATRRNVL | Mtb55 |
| VMAPATLIL | Ala 5 (small) | VLATRTLLL | Thr 4 (neutr) | VMAPRTLVL | Mtb55-VL9-hybrid |
| VMAPWTLIL | Trp 5 (big) | VLAPATLLL | Ala 5 (small) |  |  |
| VMAPDTLIL | Asp 5 (neg) | VLAPWTLLL | Trp 5 (big) |  |  |
| VMAPLTLIL | Leu 5 (neutr) | VLAPDTLLL | Asp 5 (neg) |  |  |
| VMAPRALIL | Ala 6 (small) | VLAPLTLLL | Leu 5 (neutr) |  |  |
| VMAPRWLIL | Trp 6 (big) | VLAPRALLL | Ala 6 (small) |  |  |
| VMAPRRLIL | Arg 6 (pos) | VLAPRWLLL | Trp 6 (big) |  |  |
| VMAPRDLIL | Asp 6 (neg) | VLAPRRLLL | Arg 6 (pos) |  |  |
| VMAPRILIL | Ile 6 (neutr) | VLAPRDLLL | Asp 6 (neg) |  |  |
| VMAPRPLIL | Pro 6 (kink) | VLAPRILLL | Ile 6 (neutr) |  |  |
| VMAPRTAIL | Ala 7 (small) | VLAPRPLLL | Pro 6 (kink) |  |  |
| VMAPRTWIL | Trp 7 (big) | VLAPRTALL | Ala 7 (small) |  |  |
| VMAPRTFIL | Phe 7 (big) | VLAPRTWLL | Trp 7 (big) |  |  |
| VMAPRTRIL | Arg 7 (pos) | VLAPRTFLL | Phe 7 (big) |  |  |
| VMAPRTQIL | Gln 7 (neutr) | VLAPRTRLL | Arg 7 (pos) |  |  |
| VMAPRTPIL | Pro 7 (kink) | VLAPRTDLL | Asp 7 (neg) |  |  |
| VMAPRTLAL | Ala 8 (small) | VLAPRTQLL | Gln 7 (neutr) |  |  |
| VMAPRTLGL | Gly 8 (small) | VLAPRTPLL | Pro 7 (kink) |  |  |
| VMAPRTLWL | Trp 8 (big) | VLAPRTLAL | Ala 8 (small) |  |  |
| VMAPRTLFL | Phe 8 (big) | VLAPRTLGL | Gly 8 (small) |  |  |
| VMAPRTLYL | Tyr 8 (big) | VLAPRTLWL | Trp 8 (big) |  |  |
| VMAPRTLRL | Arg 8 (pos) | VLAPRTLFL | Phe 8 (big) |  |  |
| VMAPRTLDL | Asp 8 (neg) | VLAPRTLYL | Tyr 8 (big) |  |  |
| VMAPRTLPL | Pro 8 (kink) | VLAPRTLRL | Arg 8 (pos) |  |  |
| VMAPRTLIA | Ala 9 (small) | VLAPRTLDL | Asp 8 (neg) |  |  |
| VMAPRTLIW | Trp 9 (big) | VLAPRTLPL | Pro 8 (kink) |  |  |
| VMAPRTLIF | Phe 9 (big) | VLAPRTLLA | Ala 9 (small) |  |  |
| VMAPRTLIY | Tyr 9 (big) | VLAPRTLLW | Trp 9 (big) |  |  |
|  |  | VLAPRTLLF | Phe 9 (big) |  |  |
|  |  | VLAPRTLLY | Tyr 9 (big) |  |  |
