# Supplementary material for: HLA-E/peptide complexes differentially interact with NKG2A/CD94 and T cell receptors
Source: J Immunol. 2025 Mar 14;214(4):595–605. doi: 10.1093/jimmun/vkae068 (PMC12041767; doi:10.1093/jimmun/vkae068)
Supplement: vkae068_Supplementary_Data [file vkae068_supplementary_data.zip › vkae068_Supplementary_Data/JIMMUN-24-00593-s02.docx]

Leiden, December 3, 2024

Dear Dr. Gail A. Bishop,

We are happy to hear that our manuscript ’HLA-E/peptide complexes differentially interact with NKG2A/CD94 and T cell receptors’ (ref# 24-00593-FLR) is acceptable for publication in the Journal of Immunology. We appreciate the time and effort from you and reviewers to critically read our manuscript and provide constructive feedback during the review process.

The only comment on the revised manuscript was about the truncation of Figure 3. We think that this happened during conversion of the tif file to a PDF file. We have uploaded a new version of Figure 3 to address this issue.

Changes to the manuscript are highlighted in yellow, which was only a typing error.

We look very much forward to your final decision.

Yours sincerely on behalf of all co-authors,

Dr. Simone A. Joosten

Leiden University Center for Infectious Diseases (LUCID)

Leiden University Medical Center

Albinusdreef 2, 2333 ZA, Leiden, The Netherlands

Phone: +31(0)71 526 2620

E-mail: [s.a.joosten@lumc.nl](mailto:s.a.joosten@lumc.nl)
